# Clinicopathological and prognostic significance of nestin expression in patients with breast cancer: a systematic review and meta-analysis

**DOI:** 10.1186/s12935-020-01252-5

**Published:** 2020-05-14

**Authors:** Xinyi Zhang, Changsheng Xing, Wenting Guan, Lang Chen, Kai Guo, Anze Yu, Kai Xie

**Affiliations:** 1 Department of Cardiovascular Medicine, The Second Affiliated Hospital, Zhejiang University School of Medicine, Hangzhou, 310003 China; 2 Department of Cardiovascular Medicine, Xiangya Hospital, Central South University, Changsha, 410008 China; 3 The Third Xiangya Hospital, Central South University, Changsha, 410013 China; 4 Center for Inflammation and Epigenetics, Houston Methodist Research Institute, Houston, TX 77030 USA; 5 Department of Radiology, The Affiliated Huai’an No.1 People’s Hospital of Nanjing Medical University, Huai’an, 223300 China; 6 Department of Radiology, Zhongda Hospital, Medical School of Southeast University, Nanjing, 210009 China; 7 Department of General Surgery, The Third Xiangya Hospital, Central South University, Changsha, 410013 China; 8 Department of Otolaryngology Head and Neck Surgery, Xiangya Hospital, Central South University, Changsha, 410008 China; 9 Department of Urology, Xiangya Hospital, Central South University, Changsha, 410008 China; 10 Department of General Surgery, The First Affiliated Hospital, Zhejiang University School of Medicine, Hangzhou, 310003 China; 11 Department of Neurosurgery, Xiangya Hospital, Central South University, Changsha, 410008 China

**Keywords:** Nestin, Breast cancer, Clinicopathological characteristics, Prognosis, Meta-analysis

## Abstract

**Background:**

Nestin has been revealed to promote tumorigenesis, progression, metastasis, and angiogenesis of breast cancer. Although the prognostic and clinicopathological impact of nestin expression on breast cancer patients has been assessed in several independent studies, their results remained conflicting. Therefore, we performed this meta-analysis to elucidate the prognostic and clinicopathological association of nestin expression with breast cancer.

**Methods:**

A comprehensive literature search was performed in the electronic databases PubMed, EMBASE, Web of Science, the Cochrane Library, China National Knowledge Infrastructure (CNKI), and the Wangfang Data. The statistical analysis was conducted using Stata 15.0 and Review Manager 5.3.

**Results:**

A total of 15 studies with 6066 breast cancer patients were included in this meta-analysis. Pooled results indicated that positive expression of nestin was significantly associated with reduced breast cancer-specific survival (BCSS, univariate analysis, HR = 2.11, 95% CI [1.79, 2.49], P < 0.00001; multivariate analysis, HR = 1.30, 95% CI [1.06, 1.60], P = 0.01), worse overall survival (OS, univariate analysis, HR = 1.88, 95% CI [1.31, 2.71], P = 0.0007; multivariate analysis, HR = 1.89, 95% CI [1.34, 2.67], P = 0.0003) and poorer recurrence-free survival (univariate analysis, HR = 2.60, 95% CI [1.52, 4.46], P = 0.0005), but not with distant metastasis-free survival in univariate analysis (P > 0.05). In addition, increased nestin expression was correlated with younger age, higher tumor grade, larger tumor size, positive blood vessel invasion and high vascular proliferation index, but not with lymph node metastasis or lymph vessel invasion. Nestin was preferentially expressed in invasive ductal carcinoma, triple-negative breast cancer and basal-like subtypes. Nestin expression was inversely associated with the expression of ER and PR, but not with HER-2. Conversely, nestin expression was positively correlated with the expression of basal-like markers CK5, P-cadherin and EGFR. Moreover, nestin expression was strongly associated with the presence of five basal-like profiles (BLP1-5).

**Conclusions:**

This meta-analysis revealed the prognostic value and clinicopathological significance of nestin expression in breast cancer. Nestin is an independent prognostic factor for worse BCSS and OS of breast cancer patients. Nestin is also a valuable biomarker for unfavorable clinicopathological features and tumor angiogenesis of breast cancer. Therefore, nestin is a promising therapeutic target for malignant breast cancer, especially for TNBC and basal-like phenotype.

## Background

Breast cancer (BC) is one of the most commonly diagnosed malignant tumors in women worldwide and the leading cause of cancer-related female mortality [1]. Primarily derived from epithelial cells of the mammary gland, BC is a heterogeneous disease with diverse histological patterns and biological features which results in distinct clinical behaviors [2, 3]. Histopathological classification of BC was primarily based on immunohistochemical (IHC) detection of four molecular markers implicated in growth signaling pathways: estrogen receptor (ER), progesterone receptor (PR), human epidermal growth factor receptor 2 (HER2) and proliferation marker Ki-67 [4, 5]. In accordance with different expression patterns, BC can be classified into four subtypes: luminal A (ER^+^, PR^+^, HER2^−^, low Ki-67 index), luminal B (ER^+^, PR^+^, HER2^+^ or HER2^−^, high Ki-67 index), HER2-positive (ER^−^, PR^−^, HER2^+^) and triple-negative breast cancer (TNBC, ER^−^, PR^−^, HER2^−^) [6]. Moreover, gene expression profiling can more precisely and systematically sort BC into five intrinsic molecular subtypes: luminal A, luminal B, HER2-enriched, basal-like and normal-like BC [7, 8]. Among these subtypes, basal-like subtype accounts for 15% of all invasive breast cancer and is characterized by highly aggressive behaviors, poor differentiation and lack of molecular targets for endocrine and anti-HER2 therapies [7–10]. Despite the dramatical improvements in the therapeutic strategies, including surgery, radiotherapy, chemotherapy and endocrine therapy, for fighting against BC in recent years, high rates of locoregional recurrence and metastasis of malignant breast cancer, especially TNBC and basal-like subtype, still greatly threatens the physical and mental health of women [11–15]. The relatively high cost of gene expression profiling limits its application in clinical practice [16]. Thus, identification of precise, low-cost and highly accessible biomarkers to accurately diagnose BC subtypes provides a novel approach to formulating individualized treatment regimens [17–19]. As a result, it is imperative to uncover the molecular mechanisms underlying the relapse and metastasis of malignant breast cancer and explore novel therapeutic targets to improve the management of breast cancer patients [20].

Several studies have recently targeted nestin as a promising diagnostic and prognostic marker of BC [21–28]. Nestin is a type VI intermediate filament (IF) protein encoded by the *NES* gene and originally expressed in neural progenitor cells during embryonic development [29, 30]. Nestin expression is subsequently downregulated and replaced by tissue-specific IF proteins during cell differentiation in adults [31]. Apart from neural progenitor cells, nestin expression can be detected in immature or progenitor cells in some normal tissues as well. For instance, in the normal breast tissues, nestin is expressed in the basal/myoepithelial cells of the mammary gland [32].

Extensive studies have revealed that nestin is also a putative marker for cancer stem cells (CSCs) [33–36]. CSCs are hypothetically a small subpopulation of cancer cells that possess the capacity for self-renewal as well as drive tumorigenesis and therapeutic resistance [37–39]. Aberrantly increased expression of nestin has been detected in various human malignant neoplasms, such as breast cancer [32], gliomas [35], melanomas [40], prostate cancer [36], gastrointestinal cancer [41, 42] and other cancer types [43–45]. Moreover, the prognostic value of nestin for patients with cancer has been widely validated in various solid tumors, such as epithelial ovarian cancer [46], non-small cell lung cancer [47], glioma [48, 49], etc. However, the prognostic and clinicopathological value of nestin in breast cancer patients remained controversial. Some studies have demonstrated the strong link between increased nestin expression and poor prognosis of breast cancer patients [20, 21, 23–26, 28, 50–52]. Conversely, some other studies revealed no significant association between nestin expression and survival outcomes of breast cancer patients [22, 27]. To address these discrepancies, we performed this meta-analysis to systematically determine the prognostic and clinicopathological impact of nestin on patients with breast cancer.

## Materials and methods

### Search strategies

Meta-analysis was performed in accordance with the Preferred Reporting Items for Systematic Review and Meta-Analysis (PRISMA) Statement [53]. A systematic literature search of the electronic databases PubMed, EMBASE, Web of Science, the Cochrane Library, China National Knowledge Infrastructure (CNKI), and the Wangfang Data up to October 2019 was performed, without any limitation of origin and languages. The studies were identified by a random combination of the following terms: “nestin”, “breast neoplasm OR breast tumor OR breast cancer OR breast carcinoma”, “prognosis OR survival OR outcome”. In addition, the reference lists of the retrieved studies and review articles were manually searched for potentially relevant studies.

### Selection criteria

The studies included in the present meta-analysis were randomized controlled trials (RCTs) or observational studies (case–control or cohort) that evaluated the association of nestin expression with clinicopathological features or prognosis of patients with breast cancer. Studies were eligible if they met the following criteria: (a) studies were published as original articles with full text available; (b) definitive diagnosis of breast cancer was confirmed by histopathological examination; (c) nestin expression was detected by an immunohistochemistry (IHC) method or quantitative real-time polymerase chain reaction (qRT-PCR) method based on breast cancer tissues (instead of serum or other specimens), and (d) the correlation of nestin expression with clinicopathological features or prognostic outcomes was analyzed. Studies were excluded from the analyses based on the following criteria: (a) articles were published as reviews, abstracts, case reports, letters or comments; (b) studies were not associated with the topic of the interest; (c) data were obtained from cell lines or animal models; (d) data were analyzed based on public databases; (e) data for estimating the relationship between nestin expression and survival outcomes or clinicopathological features were insufficient, and (f) data were from duplicated studies based on the same or similar patient population.

### Data extraction

Two investigators (XYZ and WTG) independently reviewed the included studies and extracted prognostic or clinicopathological data. Discrepancies in data extraction were resolved by a third investigator (KX). The following data were collected from each included study in a predefined table: the name of first author, year of publication, country, cancer types, number of patients, age, follow-up periods, detection method, cut-off value, clinicopathological parameters and prognostic outcomes (breast cancer-specific survival [BCSS], overall survival [OS], distant metastasis-free survival [DMFS], recurrence-free survival [RFS] and progression-free survival [PFS]). Since some studies displayed the survival data indirectly with a Kaplan–Meier curve, the software Engauge Digitizer version 12 (http://markummitchell.github.io/engauge-digitizer/) was applied to digitize and extract survival data. Because the cut-off value for nestin expression varied among different studies, we defined the nestin-positive group according to the original articles.

### Qualitative assessment

The methodological quality of included studies was assessed by two independent viewers (XYZ and WTG) using the Newcastle–Ottawa Quality Assessment Scale (NOS) (Additional file 1: Table S1). According to the guideline, NOS scores of ≥ 6 were determined to be high-quality studies.

### Statistical analysis

All statistical analyses were performed by using the software Review Manager 5.3.5 (Cochrane Collaboration, Copenhagen, Denmark) and STATA 15.0 (Stata Corporation, College Station, TX, USA). The odds ratio (OR) with 95% confidence interval (CI) were calculated to assess the correlation between nestin expression and clinicopathological characteristics of breast cancer. P < 0.05 were considered as statistical significance. To evaluate the prognostic effect of nestin expression on patients with breast cancer, pooled hazard ratio (HR) with 95% CI of survival outcomes were calculated. If a pooled HR is larger than 1, it reflects a worse prognosis for patients with positive nestin expression, while a pooled HR smaller than 1 represents a favorable prognosis. Heterogeneity among studies was assessed by the Chi-square (χ^2^) test and I^2^ test. When there was no significant heterogeneity (P > 0.05 or I^2^ < 50%), the fixed-effects model was employed; otherwise, the random-effects model was used. Sensitivity analyses were performed to examine the robustness of pooled data. Begg’s and Egger’s tests were conducted to assess the potential publication bias.

## Results

### Description of studies

**Fig. 1:**
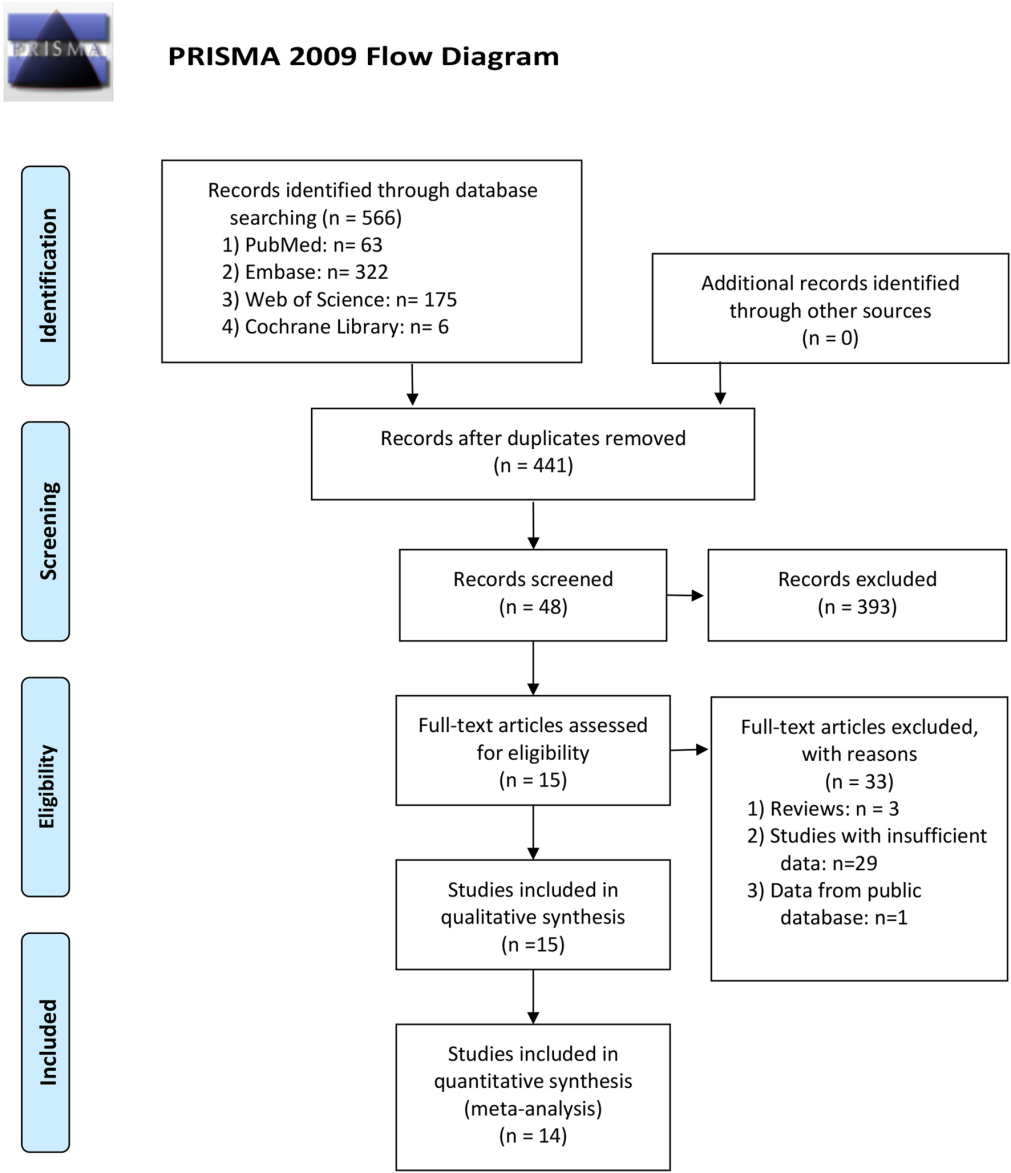
Flow diagram of the literature search and study selection in the meta-analysis

Detailed steps of literature search and study selection were shown in a flow diagram [53] (Fig. 1). A total of 566 studies were initially retrieved with search strategies described above. In line with the selection criteria, 441 articles were left after duplicated records removed. After screening the titles and abstracts of identified articles, 393 articles were excluded on account of irrelevant topics, conference abstracts or cell and animal models. The remaining 48 articles were reviewed in full text, 33 articles were excluded, including 3 reviews, 29 studies without clinicopathological or survival data, and one study based on data from the public database. A total of 15 studies with were 6066 patients eventually included in the present meta-analysis. The main characteristics of eligible studies in the qualitative and quantitative synthesis were summarized in Table 1.

**Table 1:** Characteristics of eligible studies in the qualitative and quantitative synthesis

Study	Year	Country	Cancer types	Cases/controls	Age (years)	Follow-up time	Method	Cut-off value (positive/high)	Survival outcomes	Source of data	NOS score
Asleh et al. [21]	2018	Canada	IBC	339/2895	NR	12.6 years (median)	IHC	A2 > 0	BCSS(U), BCSS(M)	Direct	8
Asleh et al. [52]	2019	Denmark	MBC	29/216	NR	NR	IHC	A2 > 0	OS(U), OS(M) PFS(U), PFS(M)	Direct	8
De Lara et al. [22]	2019	Sweden	MBC	25/110	NR	10 years	IHC	A2 > 0	OS(U), DMFS(U) OS(M), DMFS(M)	Curve Direct	8
Gao et al. [23]	2014	China	BC	42/109	32–79	NR	IHC	A1 > 0	BCSS(U)	Curve	8
Krüger et al. [24]	2017	Norway	IBC	I: 50/47 II: 35/244 III: 51/130 IV: 44/143	I: 50–69 II: 50–69 III, IV: NR	I: 13 years (median) II, III, IV: NR	IHC	At least 3 clearly positive cells	I: BCSS(U), BCSS(M) II, III, IV: NR	Direct	8
Liu et al. [26]	2010	China	DCIS, IDC	24/126	27–80	NR	IHC	A5 > 0	BCSS(U)	Curve	8
Liu et al. [25]	2012	China	DCIS, IDC	62/241	NR	7.5 years	IHC	A5 > 0	BCSS(U)	Curve	7
Meisen et al. [54]	2014	USA	BC	83/83	NR	NR	qRT-PCR	Median	DMFS(U)	Curve	7
Nowak et al. [50]	2017	Poland	IDC	109 (total)	NR	NR	IHC	Nestin^+^ MVD > 75.76 (median)	OS(U), OS(M), RFS(U)	Direct curve	7
Nowak et al. [51]	2018	Poland	IDC	39/83	NR	1–80 months	IHC	A3*B1 > 0	OS(U)	Curve	7
Parry et al. [27]	2008	UK	IBC	20/223	NR	5.2–135.3 months	IHC	A5 > 0	BCSS(U)	Curve	8
Piras et al. [28]	2011	Italy	BC	28/25	32–67	82–194 months	IHC	A6 > 0	OS(U)	Curve	8
Tampaki et al. [55]	2017	Greece	BC	26/115	NR	NR	IHC	A4 > 0	RFS(U), RFS(M)	Curve direct	7
Zhao et al. [20]	2014	China	TNBC	41/109	NR	NR	IHC	A1 > 0	OS(U)	Curve	7
Zhu et al. [56]	2009	China	DCIS, IDC	20/100	NR	NR	IHC	A5 > 0	NR	NR	–

*IBC* invasive breast cancer, *MBC* metastatic breast cancer, *BC* breast cancer, *DCIS* ductal carcinoma in situ, *IDC* invasive ductal carcinoma, *TNBC* triple-negative breast cancer, *BCSS* breast cancer-specific survival, *OS* overall survival, *PFS* progression-free survival, *DMFS* distant metastasis-free survival, *RFS* recurrence-free survival, *NR* not reported, *U* univariate analysis, *M* multivariate analysis

A: percentage of positive cells. A1: scored 0 (< 1%), 1 (1–9%), 2 (≥ 10%); A2: scored 0 (< 1%), 1 (≥ 1%); A3: scored 0 (0%), 1 (< 10%), 2 (10–50%), 3 (51–80%), 4 (> 80%); A4: scored 0 (≤ 5%), 1 (> 5%); A5: scored 0 (< 1%), 1 (1–9%), 2 (≥ 10%); A6: scored 0 (< 10%), 1 (≥ 10%)

B: intensity of staining. B1: scored 0 (absence of staining), 1 (weak staining), 2 (moderate staining), 3 (strong staining)

### Correlation between nestin expression and breast cancer-specific survival

**Fig. 2:**
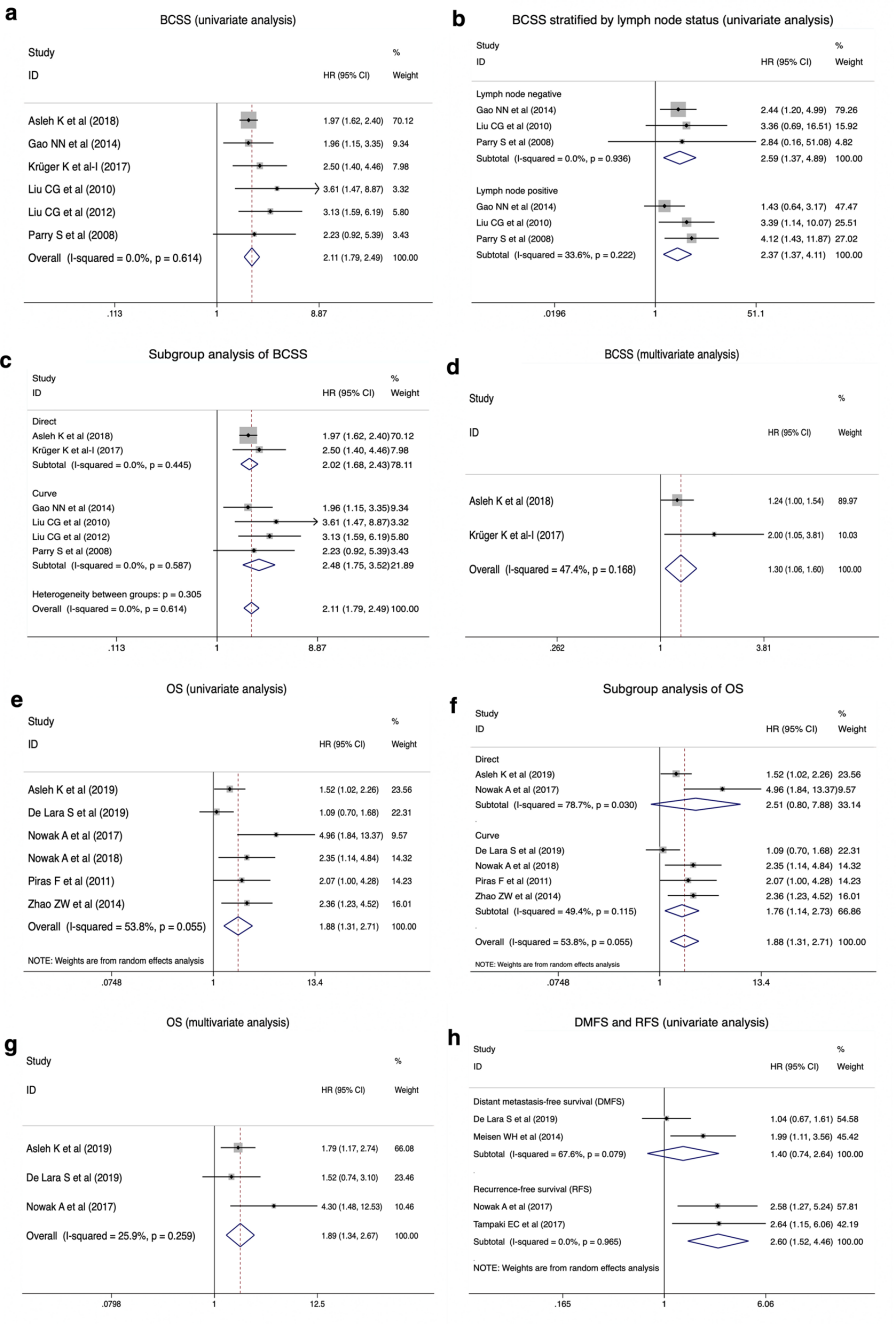
Forest plots of the correlation of nestin expression with survival outcomes. **a** The correlation of nestin expression with BCSS in univariate analysis; **b** The correlation of nestin expression with BCSS stratified by lymph node status in univariate analysis; **c** Subgroup analysis of BCSS based on univariate data stratified by source of data; **d** The correlation of nestin expression with BCSS in multivariate analysis; **e** The correlation of nestin expression with OS in univariate analysis; **f** Subgroup analysis of OS based on univariate data stratified by source of data; **g** The correlation of nestin expression with OS in multivariate analysis; **h** The correlation of nestin expression with DMFS and RFS in univariate analysis

Six studies [21, 23–27] were included in the univariate analysis of the impact of nestin expression on breast cancer-specific survival (BCSS) (Table 1). As Asleh et al. [21] described, BCSS was defined as the time from diagnosis to either death by breast cancer or last follow up. The pooled result indicated that nestin expression was significantly associated with worse BCSS (pooled HR = 2.11, 95% CI [1.79, 2.49], P < 0.00001, I^2^ = 0%) (Fig. 2a). Moreover, three studies [23, 26, 27] investigated the correlation between nestin expression and BCSS of patients stratified by lymph node status. The pooled analysis based on univariate data showed the same result both in lymph node-negative group (pooled HR = 2.59, 95% CI [1.37, 4.89], P = 0.003, I^2^ = 0%) and lymph node-positive group (pooled HR = 2.37, 95% CI [1.37, 4.11], P = 0.002, I^2^ = 33.6%) (Fig. 2b). Subgroup analysis of BCSS on the basis of univariate data was conducted and stratified in terms of source of data, including data directly obtained from articles and data calculated from Kaplan–Meier curves. Pooled results revealed that increased nestin expression in patients with breast cancer predicted worse BCSS in both subgroups (Direct data group: pooled HR = 2.02, 95% CI [1.68, 2.43], P = 0.01, I^2^ = 0%; Calculated data group: pooled HR = 2.48, 95% CI [1.75, 3.52], P = 0.01, I^2^ = 0%) (Fig. 2c). Additionally, a multivariate analysis of two studies [21, 24] demonstrated the similar result (pooled HR = 1.30, 95% CI [1.06, 1.60], P = 0.01, I^2^ = 47.4%) (Table 1, Fig. 2d).

### Correlation between nestin expression and overall survival

A pooled analysis of six studies [20, 22, 28, 50–52] reported overall survival (OS) data using univariate analysis (Table 1). With slight heterogeneity (P = 0.055, I^2^ = 53.8%), a random-effects model showed increased nestin expression in patients with breast cancer predicted reduced OS (pooled HR = 1.88, 95% CI [1.31, 2.71], P = 0.0007) (Fig. 2e). Stratified by source of data, subgroup analysis of OS based on univariate data demonstrated that nestin expression was significantly correlated with OS in calculated data group (pooled HR = 1.76, 95% CI [1.14, 2.73], P = 0.01, I^2^ = 49.4%), but not with OS in direct data group due to substantial heterogeneity between two included studies (pooled HR = 2.51, 95% CI [0.80, 7.88], P = 0.12, I^2^ = 78.7%). Furthermore, a pooled analysis of three studies [22, 50, 52] based on multivariate data also exhibited the same result (pooled HR = 1.89, 95% CI [1.34, 2.67], P = 0.0003, I^2^ = 26%) (Table 1, Fig. 2g).

### Correlation between nestin expression and other survival outcomes

Two studies [22, 54] including 301 patients, investigated the correlation between nestin expression and distant metastasis-free survival (DMFS) based on univariate data (Table 1). As Lara et al. [22] described, DMFS refers to the time from the initial diagnosis of the primary breast carcinoma to distant metastasis. With moderate heterogeneity (P = 0.079, I^2^ = 67.6%), a random-effects model showed no significant association between nestin expression and DMFS (pooled HR = 1.40, 95% CI [0.74, 2.64], P = 0.30) (Fig. 2h). Moreover, the study by Lara et al. [22] revealed that nestin is neither an independent prognostic factor for DMFS in multivariate analysis (HR = 1.52, 95% CI [0.75, 3.06], P = 0.25).

Another two studies [50, 55] with 249 patients were included in the univariate analysis of the correlation of nestin expression with recurrence-free survival (RFS) (Table 1). RFS is defined as the time from the initial diagnosis of the primary breast carcinoma to recurrence. The pooled result demonstrated that increased nestin expression predicts poorer RFS (pooled HR = 2.60, 95% CI [1.52, 4.46], P = 0.0005, I^2^ = 0%) (Fig. 2h). However, the study by Tampaki et al. [55] reported that nestin is not an independent prognostic factor for RFS in multivariate analysis (HR = 1.238, 95% CI [0.512, 2.989], P = 0.636).

In addition, Asleh et al. [52] also reported the impact of nestin expression on progression-free survival (PFS). Increased expression of nestin was associated with reduced PFS based on univariate data (HR = 1.65, 95% CI [1.04, 2.63], P = 0.03). However, no significant association was noted between nestin expression and PFS in multivariate analysis (HR = 1.26, 95% CI [0.75, 2.11], P = 0.39).

### Correlation between nestin expression and clinicopathological parameters

**Fig. 3:**
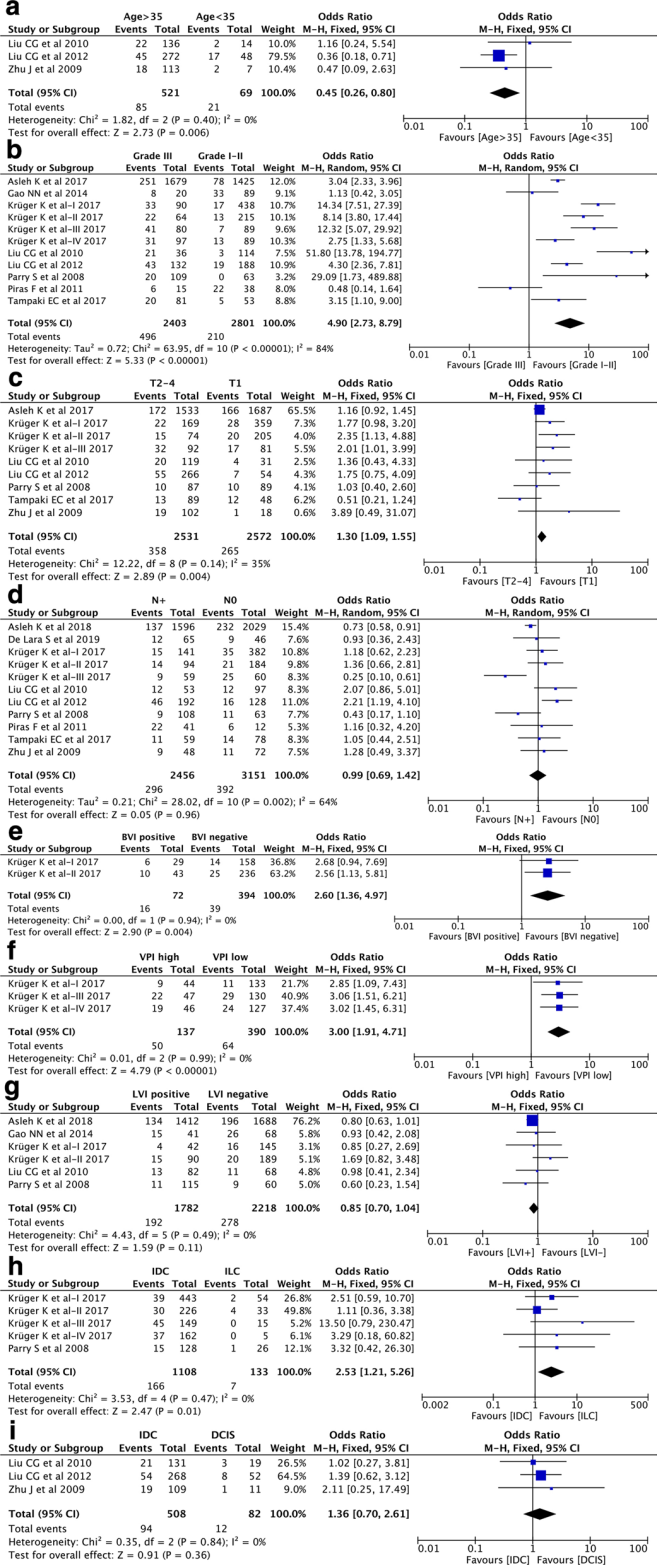
Forest plots of the correlation between nestin expression and clinicopathological parameters. **a** Age (age > 35 vs. age < 35); **b** TNM stage (grade III vs. grade I–II); **c** Tumor size (T_2–4_ vs. T_1_); **d** Lymph node metastasis (N_+_ vs. N_0_); **e** BVI (positive vs. negative); **f** VPI (high vs. low); **g** LVI (positive vs. negative); **h** IDC vs. ILC; **i** IDC vs. DCIS

A total of three studies [25, 26, 56] with 590 patients investigated the association between nestin expression and age. Pooled result revealed that nestin was preferentially expressed in patients younger than 35 years old (Age > 35 vs. Age < 35, pooled OR = 0.45, 95% CI [0.26, 0.80], P = 0.006, I^2^ = 0%) (Fig. 3a). Additionally, the study by Asleh et al. [21] including 3234 patients demonstrated that nestin expression rate was higher in patients younger than 61 years old (Age > 60 vs. Age ≤ 60, OR = 0.57, 95% CI [0.45, 0.72], P < 0.00001). These results indicated nestin was closely associated with a younger age for the onset of BC.

To confirm the correlation between nestin expression and TMN staging (histological grade, tumor size and lymph node status), a pooled analysis of thirteen datasets from ten studies [21–28, 55, 56] showed that nestin expression was significantly associated with higher tumor grade (grade III vs. grade I–II, pooled OR = 4.90, 95% CI [2.73, 8.79], P < 0.00001, I^2^ = 84%) (Fig. 3b) and larger tumor size (T > 2 cm vs. T < 2 cm, pooled OR = 1.30, 95% CI [1.09, 1.55], P = 0.004, I^2^ = 35%) (Fig. 3c), but not with lymph node metastasis (N_+_ vs. N_0_, pooled OR = 0.99, 95% CI [0.69, 1.42], P = 0.96, I^2^ = 64%) (Fig. 3d).

Nestin has recently drawn attention as a marker for tumor angiogenesis. Eight datasets from five studies [21, 23, 24, 26, 27] were included in investigating the correlation of nestin expression with three angiogenesis-related variables blood vessel invasion (BVI), lymph vessel invasion (LVI) and vascular proliferation index (VPI). As Krüger et al. [24] described, VPI was defined as the proportion of vessels with proliferating endothelial cells. Pooled results demonstrated that nestin expression was significantly associated with positive BVI (pooled OR = 2.60, 95% CI [1.36, 4.97], P = 0.004, I^2^ = 0%) (Fig. 3e) and high VPI (pooled OR = 3.00, 95% CI [1.91, 4.71], P < 0.00001, I^2^ = 0%) (Fig. 3f), but not with LVI (positive vs. negative, pooled OR = 0.85, 95% CI [0.70, 1.04], P = 0.11, I^2^ = 0%) (Fig. 3g).

Invasive ductal carcinoma (IDC) and invasive lobular carcinoma (ILC) are the most common histological types of invasive breast cancer (IBC) [57]. Five datasets from two studies [24, 27] investigated the differential expression of nestin between IDC and ILC. Pooled result revealed an increased expression level of nestin in IDC when compared with ILC (pooled OR = 2.53, 95% CI [1.21, 5.26], P = 0.01, I^2^ = 0%) (Fig. 3h). Moreover, three studies [25, 26, 56] were included in the comparison of nestin expression between IDC and ductal carcinoma in situ (DCIS). It revealed that there was no significant difference in nestin expression between IDC and DCIS (IDC vs. DCIS, pooled OR = 1.36, 95% CI [0.70, 2.61], P = 0.36, I^2^ = 0%) (Fig. 3i).

### Correlation between nestin expression and immunohistochemical markers

**Fig. 4:**
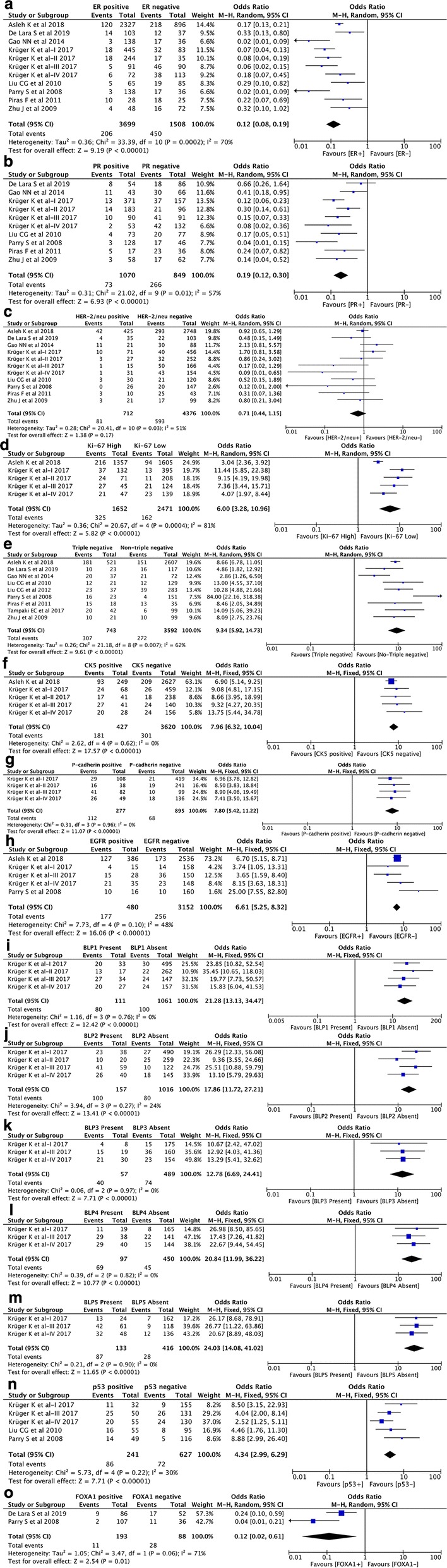
Forest plots of the correlation between nestin expression and IHC markers. **a** ER (positive vs. negative); **b** PR (positive vs. negative); **c** HER2 (positive vs. negative); **d** Ki-67 (high vs. low); **e** TNBC vs. Non-TNBC; **f** CK5 (positive vs. negative); **g** P-cadherin (positive vs. negative); **h** EGFR (positive vs. negative); **i** BLP1 (present vs. absent); **j** BLP2 (present vs. absent); **k** BLP3 (present vs. absent); **l** BLP4 (present vs. absent); **m** BLP5 (present vs. absent); **n** p53 (positive vs. negative); **o** FOXA1 (positive vs. negative)

A total of 11 datasets from 8 studies [21–24, 26–28, 56] investigated the correlation of nestin expression with the expression of ER, PR, HER-2 and Ki-67. The pooled results showed that nestin expression was negatively associated with the expression of ER (pooled OR = 0.12, 95% CI [0.08, 0.19], P < 0.00001, I^2^ = 70%) (Fig. 4a) and PR (pooled OR = 0.19, 95% CI [0.12, 0.30], P < 0.00001, I^2^ = 57%) (Fig. 4b). Besides, nestin expression was positively correlated with high expression of cell proliferation marker Ki-67 (pooled OR = 6.00, 95% CI [3.28, 10.96], P < 0.00001, I^2^ = 81%) (Fig. 4d). However, there was no significant association between the expression of nestin and HER-2 status (pooled OR = 0.71, 95% CI [0.44, 1.15], P = 0.17, I^2^ = 51%) (Fig. 4c). More importantly, nine studies with 4335 patients [21–23, 25–28, 55, 56] compared the nestin expression between triple negative breast cancer (TNBC) and non-triple negative breast cancer. With moderate heterogeneity (P = 0.007, I^2^ = 62%), the pooled analysis in random-effects model revealed nestin was preferentially expressed in TNBC than that in non-TNBC (pooled OR = 9.34, 95% CI [5.92, 14.73], P < 0.00001) (Fig. 4e). Additionally, the correlation between the expression nestin and some other biomarkers, such as p53 and forkhead box protein A1 (FOXA1), was also reported in several studies [22, 24, 26, 27]. Nestin expression revealed significant association with positive p53 nuclear expression (pooled OR = 4.34, 95% CI [2.99, 6.29], P < 0.00001, I^2^ = 30%) (Fig. 4n) and negative expression of FOXA1 (pooled OR = 0.12, 95% CI [0.02, 0.61], P = 0.01, I^2^ = 71%) (Fig. 4o).

Three studies with six datasets [21, 24, 27] were included in the analysis of the association of nestin expression with basal-like markers cytokeratin 5 (CK5), P-cadherin and epidermal growth factor receptor (EGFR). The pooled results demonstrated that nestin expression was significantly correlated with the positive expression of CK5 (pooled OR = 7.96, 95% CI [6.32, 10.04], P < 0.00001, I^2^ = 0%) (Fig. 4f), P-cadherin (pooled OR = 7.80, 95% CI [5.42, 11.22], P < 0.00001, I^2^ = 0%) (Fig. 4g), and EGFR (pooled OR = 6.61, 95% CI [5.25, 8.32], P < 0.00001, I^2^ = 48%) (Fig. 4h). Moreover, four series (series I–IV) from the study by Krüger et al. [24] with 1172 breast cancer patients also investigated the correlation of nestin expression with basal-like profiles (BLP). As Krüger et al. [24] described, five basal-like profiles (BLPs) were defined based on the combination of different IHC markers (BLP1: ER^−^, HER2^−^, CK5+; BLP2: ER^−^, HER2^−^, P-cadherin+; BLP3: ER^−^, HER2^−^, EGFR+; BLP4: ER^−^, HER2^−^, CK5+ and/or EGFR+; BLP5: ER^−^, HER2^−^, CK5+ and/or P-cadherin+ and/or EGFR+). Among these five BLPs, BLP4 is regarded as a core basal phenotype (CBP). The pooled results revealed that nestin expression was strongly correlated with the presence of BLPs (BLP1: pooled OR = 21.28, 95% CI [13.13, 34.47], P < 0.00001, I^2^ = 0%; BLP2: pooled OR = 17.86, 95% CI [11.72, 27.21], P < 0.00001, I^2^ = 24%; BLP3: pooled OR = 12.78, 95% CI [6.69, 24.41], P < 0.00001, I^2^ = 0%; BLP4: pooled OR = 20.84, 95% CI [11.99, 36.22], P < 0.00001, I^2^ = 0%; BLP5: pooled OR = 24.03, 95% CI [14.08, 41.02], P < 0.00001, I^2^ = 0%) (Fig. 4i–m). Furthermore, the study by Asleh et al. including 3128 patients [21] indicated that nestin was predominantly expressed in core basal subtype than that in non-core basal subtype (OR = 10.59, 95% CI [8.08, 13.88], P < 0.00001).

### Correlation between nestin expression and adjuvant systemic therapies

**Fig. 5:**
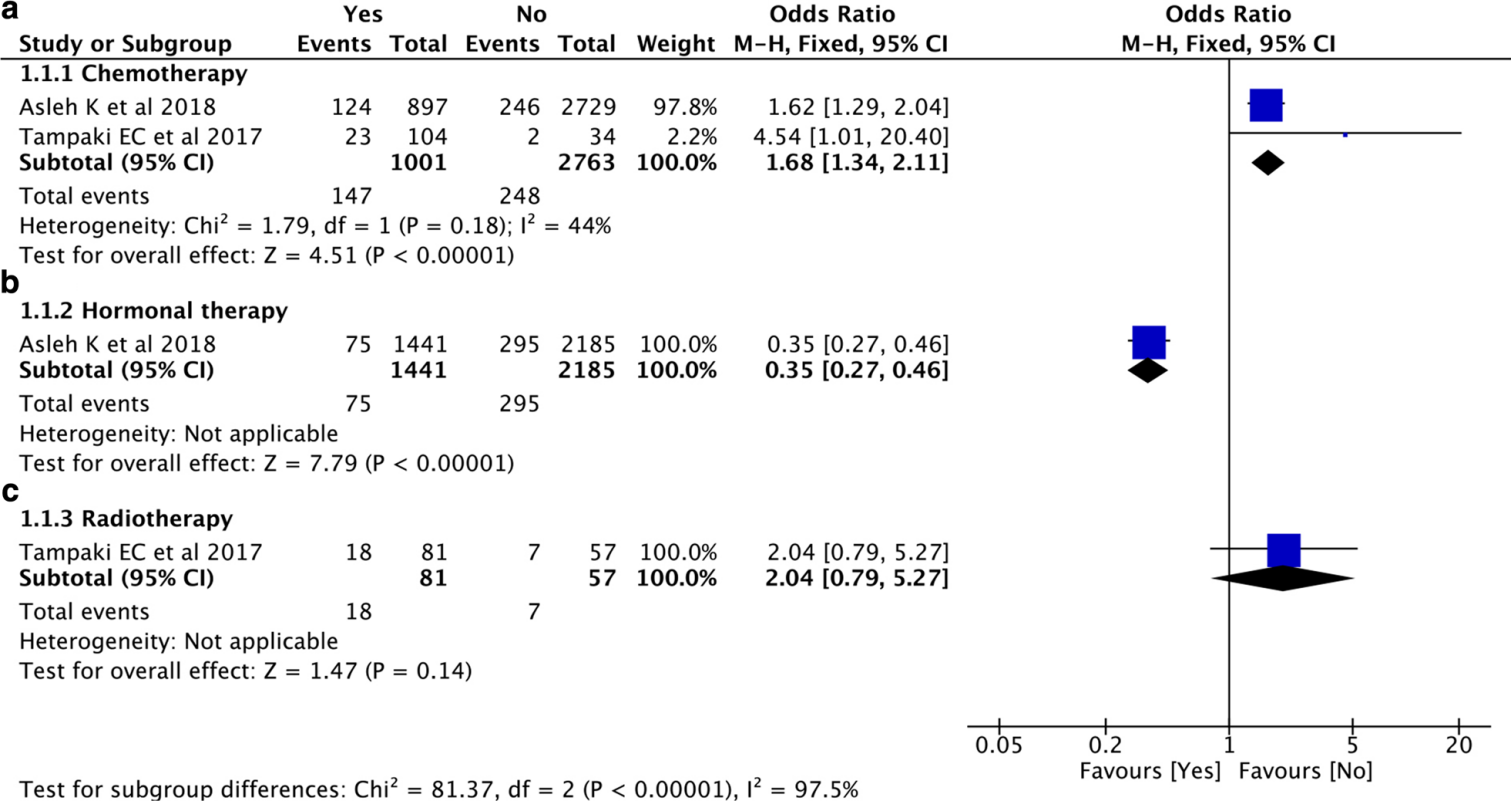
Forest plots of the correlation between nestin expression and adjuvant systemic therapies. **a** Chemotherapy; **b** Hormonal therapy; **c** Radiotherapy

Two studies [21, 55] with 3764 patients investigated the correlation between nestin expression and chemotherapy. Pooled result indicated an increased expression level of nestin in patients treated with chemotherapy than that in patients without receiving chemotherapy (pooled OR = 1.68, 95% CI [1.34, 2.11], P < 0.00001, I^2^ = 44%) (Fig. 5a). Besides, the study by Asleh et al. [21] also revealed that expression level of nestin was down-regulated in patients treated with hormonal therapy (Tamoxifen) than that in patients without receiving hormonal therapy (OR = 0.35, 95% CI [0.27, 0.46], P < 0.00001) (Fig. 5b). However, the study by Tampaki et al. [55] showed no significant difference in nestin expression between patients with radiotherapy than that in patients without receiving radiotherapy (OR = 2.04, 95% CI [0.79, 5.27], P = 0.14) (Fig. 5c).

### Sensitivity analysis and publication bias

**Fig. 6:**
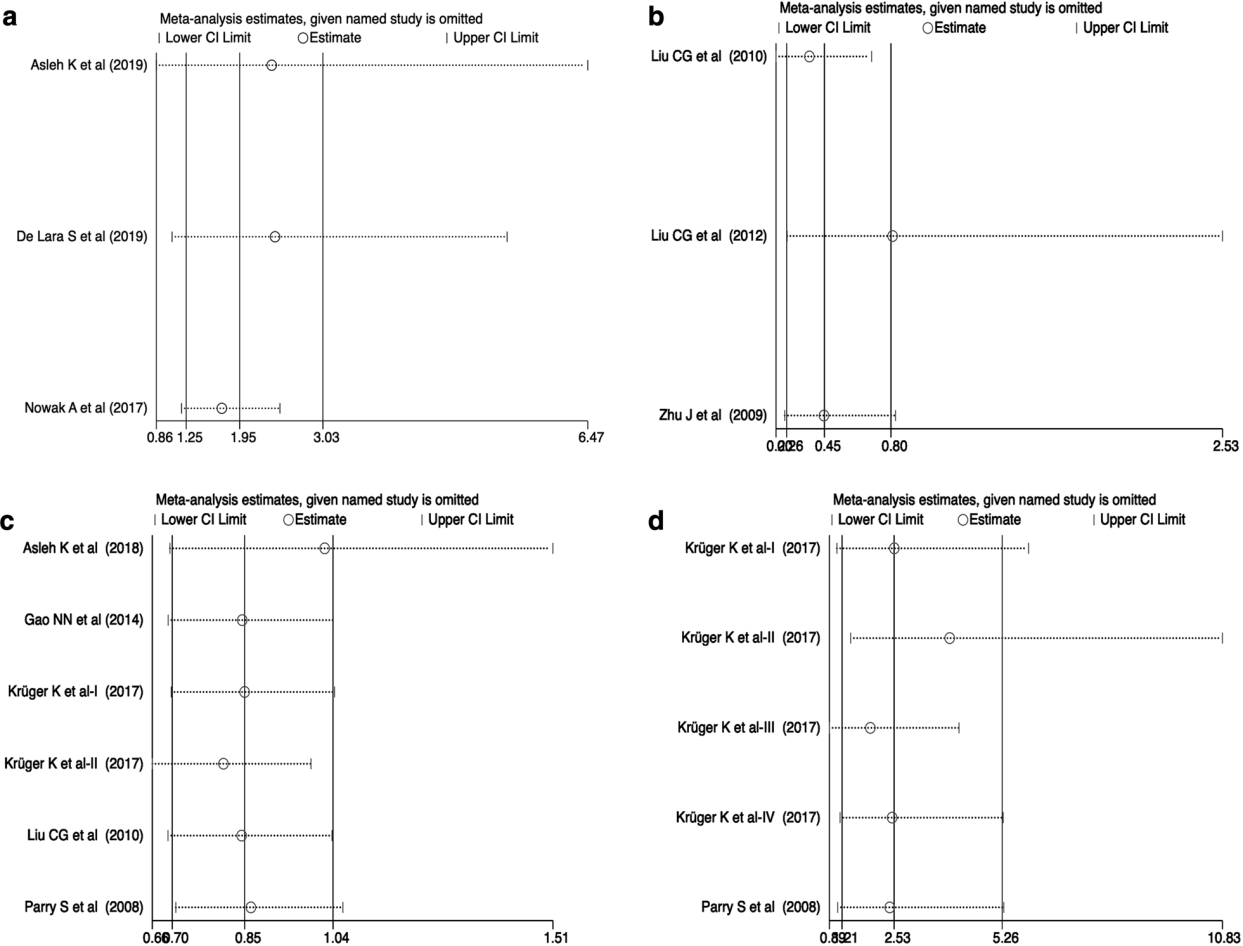
Sensitivity analysis. **a** OS in multivariate analysis; **b** Age (age > 35 vs. age < 35); **c** LVI (positive vs. negative); **d** IDC vs. ILC

Sensitivity analysis was performed to validate the stability of the pooled studies (Fig. 6, Additional file 1: Fig. S1). As shown in Table 2, no individual study could statistically significantly alter the combined results of survival outcomes and clinicopathological parameters but the study by Asleh et al. [52], the study by Liu et al. [25] and Series II from the study by Krüger et al. [24], which altered the pooled results of the correlation of nestin expression with OS (multivariate analysis), age, and LVI, respectively (Fig. 6a–c). Besides, Series III from the study by Krüger et al. [24] can affect the pooled OR of nestin expression between IDCs and ILCs (Fig. 6d). More eligible studies should be added in these pooled analyses to draw stable conclusions.

**Table 2:** Sensitivity analysis and publication bias

	Sensitivity analysis		Publication bias (P value)	
	HR/OR fluctuation	95% CI fluctuation	Begg’s test	Egger’s test
Nestin expression and survival outcomes				
BCSS(U)	2.061–2.488	1.741–3.357	0.060	0.065
BCSS(M)	1.240–2.000	1.000–3.810	1.000	–
OS(U)	1.648–2.117	1.202–3.386	0.260	0.018
OS(M)	1.715–2.362	0.862–6.474	1.000	0.536
DMFS(U)	1.040–1.993	0.674–3.564	1.000	–
RFS(U)	2.575–2.640	1.150–6.061	1.000	–
Nestin expression and clinicopathological parameters				
Age (> 35 vs. < 35)	0.375–0.809	0.200–2.529	1.000	0.437
TNM stage (III vs. I-II)	4.051–5.924	2.337–10.828	0.876	0.386
T stage (T_2–4_ vs. T_1_)	1.256–1.568	1.046–2.097	0.917	0.340
N stage (N_+_ vs. N_0_)	0.893–1.114	0.637–1.586	0.755	0.358
IDC vs. DCIS	1.280–1.477	0.428–3.928	1.000	0.817
IDC vs. ILC	1.927–3.935	0.890–10.835	0.221	0.056
LVI (positive vs. negative)	0.806–1.021	0.656–1.505	1.000	0.484
BVI (positive vs. negative)	2.558–2.683	0.936–7.689	1.000	–
VPI (high vs. low)	2.958–3.043	1.650–5.302	0.296	0.066
Nestin expression and immunohistochemical markers				
ER (positive vs. negative)	0.107–0.134	0.064–0.208	0.243	0.306
PR (positive vs. negative)	0.167–0.218	0.105–0.341	0.474	0.455
HER2 (positive vs. negative)	0.610–0.826	0.321–1.268	0.013	0.068
Ki-67 (high vs. low)	4.998–7.542	2.807–14.262	0.462	0.059
TNBC vs. Non-TNBC	7.898–10.775	5.246–18.386	0.348	0.657
CK5 (positive vs. negative)	7.605–9.786	5.983–14.333	0.462	0.023
P-cadherin (positive vs. negative)	7.440–8.244	4.945–12.964	0.308	0.123
EGFR (positive vs. negative)	6.306–7.034	3.962–10.244	1.000	0.888
BLP1 (present vs. absent)	19.543–23.784	11.296–41.447	1.000	0.629
BLP2 (present vs. absent)	15.339–20.293	9.432–33.109	0.308	0.280
BLP3 (present vs. absent)	12.265–13.134	4.821–31.205	0.296	0.253
BLP4 (present vs. absent)	19.715–23.878	9.672–48.403	0.296	0.468
BLP5 (present vs. absent)	22.230–26.590	11.294–53.144	1.000	0.683
p53 (positive vs. negative)	3.906–5.397	2.619–8.421	0.221	0.055
FOXA1 (positive vs. negative)	0.043–0.241	0.009–0.593	1.000	–

**Fig. 7:**
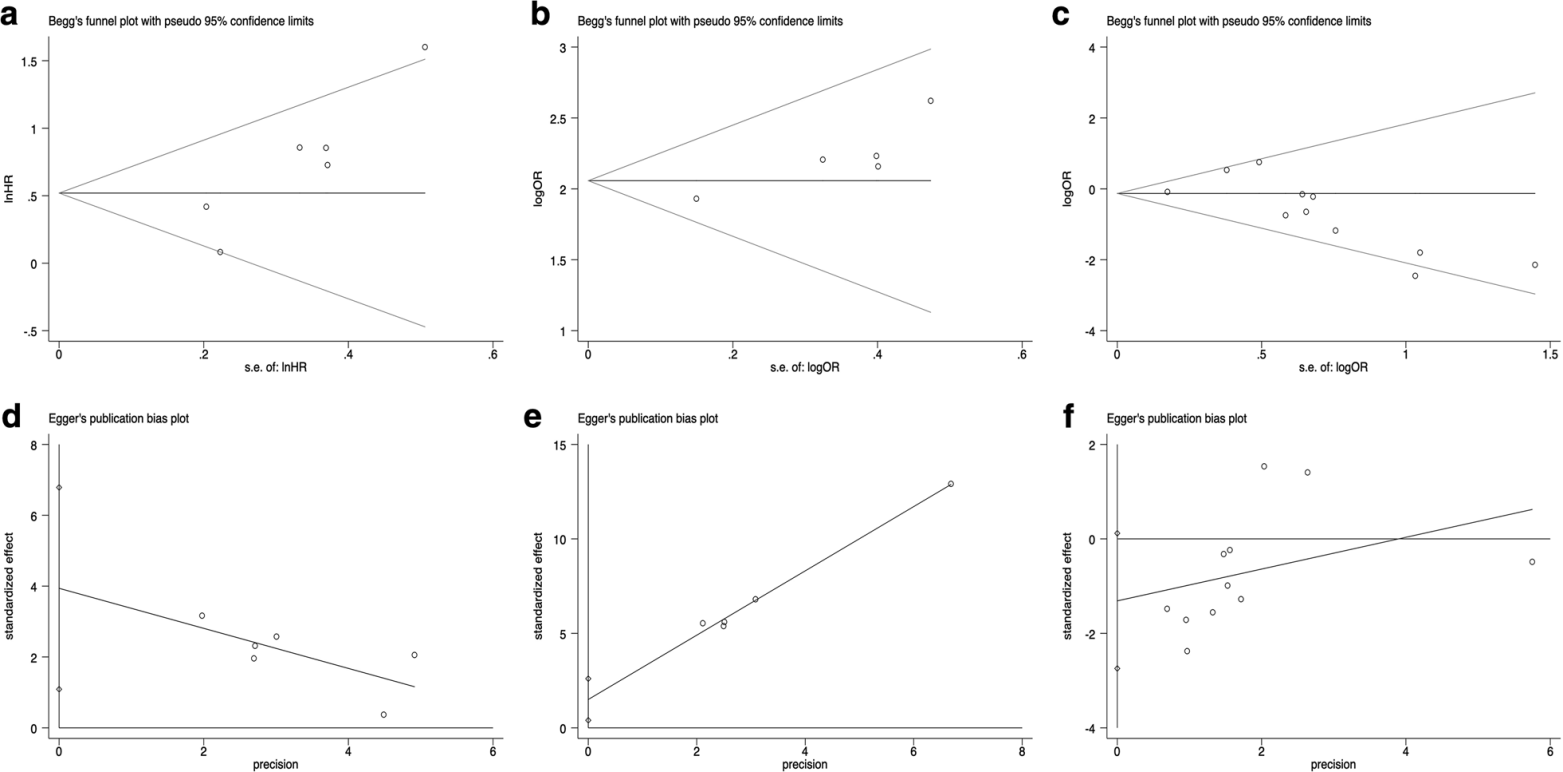
Publication bias. **a**, **d**: The correlation of nestin expression with OS in univariate analysis (**a** Begg’s test; **d** Egger’s test); **b**, **e** The correlation of nestin expression with HER2 status (**b** Begg’s test; **e** Egger’s test); **c**, **f**: The correlation of nestin expression with CK5 status (**c** Begg’s test; **f** Egger’s test)

**Fig. 8:**
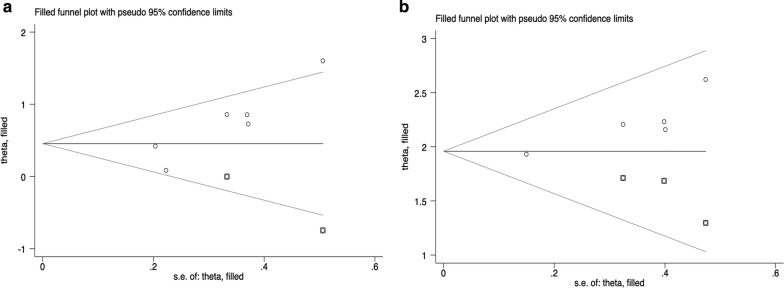
Trim and fill analysis. **a** The correlation of nestin expression with OS in univariate analysis; **b** The correlation of nestin expression with CK5 status

Potential publication bias of survival outcomes and clinicopathological parameters was evaluated using Begg’s test (Fig. 7a–c, Additional file 2: Fig. S2) and Egger’s test (Fig. 7d–f, Additional file 3: Fig. S3). As shown in Table 2, P values assessed by Begg’s test and Egger’s test were all greater than 0.05 except the evidence of significant publication bias (P < 0.05) in three pooled studies (Fig. 7). Therefore, the trim and fill method was utilized to evaluate the potential impacts of publication bias. For the pooled analysis of the association of nestin expression with OS of breast cancer (univariate analysis, Egger’s test, P = 0.018), a filled funnel plot was generated by trim and fill analysis including two imputed studies, and the meta-analysis incorporating these two imputed studies demonstrated the similar result (univariate analysis, adjusted HR = 1.575, 95% CI [1.089–2.279], P = 0.016) (Fig. 8a). Similarly, trim and fill analysis including three imputed studies generated a symmetrical funnel plot for the pooled analysis of the association between the expression of nestin and CK5 (Egger’s test, P = 0.023), and the meta-analysis incorporating these three imputed studies demonstrated the semblable result (adjusted OR = 7.091, 95% CI [5.777–8.704], P < 0.001) (Fig. 8b). Despite a significant publication bias in the analysis of the association between nestin expression and HER2 status (Begg’s test, P = 0.013), trim and fill analysis revealed that no trimming was performed and thus pooled data remained unchanged. In conclusion, the results of the three pooled studies were robust in spite of significant publication bias.

## Discussion

Nestin, initially identified as a neural stem cell marker, has recently been implicated as a positive regulator of proliferation, survival, and invasiveness of breast CSCs via the Wnt/β-catenin pathway [20, 58]. Upregulated expression of nestin promoted the tumorigenicity of breast CSCs, whereas inhibition of nestin expression can significantly induce CSC cycle arrest at G2/M phases and promote CSC apoptosis [20]. Silencing nestin expression can notably suppress the epithelial-mesenchymal transition (EMT) process that is closely related to the invasiveness of breast CSCs [20, 26, 59, 60]. Therefore, it is imperative to elucidate the clinical implication of nestin in breast cancer patients.

Previous studies investigated the potential prognostic and clinicopathological association of nestin expression with breast cancer patients. However, the eligible studies included in this meta-analysis were diversified and their results were contradictory. We first conducted the present meta-analysis to systematically analyze the prognostic impact of nestin expression on breast cancer patients. Pooled results demonstrated that positive expression of nestin predicted shorter BCSS and reduced OS of breast cancer patients in both univariate and multivariate analyses. Therefore, nestin is an independent factor for worse BCSS and OS of breast cancer patients. Besides, nestin-positive expression was also associated with RFS in univariate analysis, but not with DMFS based on univariate data. Of note, because only limited studies were available for the pooled analyses of the prognostic impact of nestin expression on DMFS and RFS, it was highly possible to draw unreliable and biased conclusions. Therefore, more studies to investigate the relationship between nestin expression and survival outcomes of breast cancer patients are warranted.

We then evaluated the correlation between nestin expression and clinicopathological characteristics of breast cancer patients in this meta-analysis. Nestin positivity was closely associated with earlier age for the onset of disease, higher histological grade, larger tumor size, and IDC, but not with lymph node metastasis. Nestin expression was correlated with ER negativity, PR negativity, high Ki-67 index, and positive p53 nuclear expression, but not with HER-2 status. In addition, positive expression of nestin was strongly correlated with three basal markers (CK5, P-cadherin, and EGFR) and five basal-like profiles (BLP1–BLP5). More importantly, nestin was predominantly expressed in malignant breast cancer, especially in TNBC and basal-like phenotype. Nestin expression was also revealed to be predominant in patients treated with chemotherapy and be down-regulated in patients receiving hormonal therapy. These results are in accordance with the negative correlation of nestin expression with hormonal receptor expression. In view of results mentioned above, nestin expression was correlated with unfavorable clinicopathological features in breast cancer patients. Enhanced expression of nestin is a promising indicator for the malignancy of breast cancer. Of note, although Li et al. [32] evaluated nestin as a selective marker of basal epithelial breast tumors, it was reported that high levels of nestin expression were also detected in non-TNBCs and non-core basal subtypes, such as in a small group of luminal subtypes according to some studies [21–23, 25–28, 55, 56]. As a result, nestin is not actually an optimized predictor of TNBC or basal-like phenotype.

Tumor angiogenesis on the basis of new vascular network formation plays a vital role in the progression and invasiveness of breast cancer [61, 62]. Apart from participation in the angiogenesis of wound healing and tissue repair in various normal tissues, nestin expression has also been implicated in tumor angiogenesis [31, 63]. Nestin-positive breast cancer stem/progenitor cells can differentiate into endothelial cells which would participate in tumor growth and vascularization [64]. Accumulated evidence suggested that nestin expression in the blood vessels is basically localized to the newly formed endothelial cell of tumor vessels [65, 66]. In this meta-analysis, we validated that nestin expression was significantly associated with positive blood vessel invasion and high vascular proliferation index in BC, but not with lymph vessel invasion. Besides, according to the study by Nowak et al. [50], increased nestin-positive microvessel density (Nestin^+^MVD) was found to be significantly associated with a shorter OS, earlier relapse, higher histological grade, and TNBCs. In consequence, nestin can be a potential angiogenesis-specific marker in BC.

Several studies also assessed the potential diagnostic and prognostic value of co-expression of nestin and some other biomarkers in breast cancer. Asleh et al. [52, 67] revealed that IHC detection of nestin and inositol polyphosphate-4-phosphatase (INPP4b) as an optimized panel of markers can be a more specific indicator for the basal-like subtype of BC regardless of ER status. Nestin positivity or loss of INPP4b (Nestin^+^ or INPP4b^−^) is an independent prognostic for BCSS of basal-like cases with weak ER positivity [67]. Liu et al. [25] found that co-expression of nestin and octamer-binding transcription factor 4 (Oct-4) was an independent prognostic factor for breast cancer (OR = 10.114, 95% CI [1.632, 62.699], P = 0.013). Nestin/Oct-4 co-expression was significantly correlated with poor prognosis, younger age, lymph node metastasis, and TNBCs [25]. Besides, a study by Rögelsperger et al. [68] demonstrated that abundant expression of G-protein-coupled receptor for melatonin (MT1) was frequently observed in advanced breast cancer specimens. Previous studies showed that MT1 activation by melatonin can induce anti-tumor effects and inhibit the cell growth and metastasis of breast cancer [69, 70]. However, a high level of MT1 expression was also detected in breast cancer cells and was involved in tumor initiation and progression of breast cancer on basis of its activation independent of melatonin [71, 72]. Co-localization of nestin and MT1 in invasive breast cancer was associated with worse prognosis and advanced histological stage [68]. As a consequence, IHC detection of co-expression of nestin and relevant biomarkers provides a novel approach to more precisely predicting the prognosis of breast cancer.

There were some potential limitations to this meta-analysis. First, partial survival data were extracted from Kaplan–Meier curves in some studies, which are less dependable than data directly obtained from articles. Second, the criterion of positive nestin expression was verified among eligible studies, which may result in the heterogeneity of studies. Finally, only limited studies were included into the multivariate analysis of the correlation between nestin expression and survival outcomes, which may draw biased conclusions. This warrants further studies added into the meta-analysis of the prognostic impact of nestin expression on survival outcomes to obtain robust and reliable results. Despite the limitations mentioned above, the present meta-analysis still revealed the prognostic value of nestin expression in breast cancer patients.

## Conclusions

In summary, this meta-analysis revealed the prognostic value and clinicopathological significance of nestin expression in breast cancer. Nestin is an independent prognostic factor for worse BCSS and OS of breast cancer patients. Besides, nestin is also a valuable biomarker for unfavorable clinicopathological features and tumor angiogenesis of breast cancer. More prospective studies with multivariate analysis to evaluate nestin as a therapeutic target for malignant breast cancer, especially for TNBC and basal-like phenotype, are warranted.

## Supplementary information

**Additional file 1: Table S1.** Newcastle–Ottawa Scale for assessing the quality of studies in quantitative analysis.

**Additional file 2: Fig. S1.** Sensitivity analysis. (1) BCSS (univariate analysis); (2) BCSS (multivariate analysis); (3) OS (univariate analysis); (4) OS (multivariate analysis); (5) DMFS (univariate analysis); (6) RFS (univariate analysis); (7) TNM stage (grade III vs. grade I–II); (8) Tumor size (T_2–4_ vs. T_1_); (9) Lymph node metastasis (N_+_ vs. N_0_); (10) IDC vs. DCIS; (11) BVI (positive vs. negative); (12) VPI (high vs. low); (13) ER (positive vs. negative); (14) PR (positive vs. negative); (15) HER2 (positive vs. negative); (16) Ki-67 (high vs. low); (17) TNBC vs. Non-TNBC; (18) CK5 (positive vs. negative); (19) P-cadherin (positive vs. negative); (20) EGFR (positive vs. negative); (21) BLP1 (present vs. absent); (22) BLP2 (present vs. absent); (23) BLP3 (present vs. absent); (24) BLP4 (present vs. absent); (25) BLP5 (present vs. absent); (26) p53 (positive vs. negative); (27) FOXA1 (positive vs. negative).

**Additional file 3: Fig. S2.** Begg’s test. (1) BCSS (univariate analysis); (2) BCSS (multivariate analysis); (3) OS (multivariate analysis); (4) DMFS (univariate analysis); (5) RFS (univariate analysis); (6) Age (age > 35 vs. age < 35); (7) TNM stage (grade III vs. grade I–II); (8) Tumor size (T_2–4_ vs. T_1_); (9) Lymph node metastasis (N_+_ vs. N_0_); (10) IDC vs. DCIS; (11) IDC vs. ILC; (12) LVI (positive vs. negative); (13) BVI (positive vs. negative); (14) VPI (high vs. low); (15) ER (positive vs. negative); (16) PR (positive vs. negative); (17) Ki-67 (high vs. low); (18) TNBC vs. Non-TNBC; (19) P-cadherin (positive vs. negative); (20) EGFR (positive vs. negative); (21) BLP1 (present vs. absent); (22) BLP2 (present vs. absent); (23) BLP3 (present vs. absent); (24) BLP4 (present vs. absent); (25) BLP5 (present vs. absent); (26) p53 (positive vs. negative); (27) FOXA1 (positive vs. negative).

**Additional file 4: Fig. S3.** Egger’s test. (1) BCSS (univariate analysis); (2) OS (multivariate analysis); (3) Age (age > 35 vs. age < 35); (4) TNM stage (grade III vs. grade I–II); (5) Tumor size (T_2–4_ vs. T_1_); (6) Lymph node metastasis (N_+_ vs. N_0_); (7) IDC vs. DCIS; (8) IDC vs. ILC; (9) LVI (positive vs. negative); (10) VPI (high vs. low); (11) ER (positive vs. negative); (12) PR (positive vs. negative); (13) Ki-67 (high vs. low); (14) TNBC vs. Non-TNBC; (15) P-cadherin (positive vs. negative); (16) EGFR (positive vs. negative); (17) BLP1 (present vs. absent); (18) BLP2 (present vs. absent); (19) BLP3 (present vs. absent); (20) BLP4 (present vs. absent); (21) BLP5 (present vs. absent); (22) p53 (positive vs. negative).

## Data Availability

The datasets used in this study are available from the corresponding author upon reasonable request.

## Abbreviations

BC: Breast cancer
BCSS: Breast cancer-specific survival
BLP: Basal-like profiles
BVI: Blood vessel invasion
CBP: Core basal phenotype
CI: Confidence interval
CK5: Cytokeratin 5
CSC: Cancer stem cell
DCIS: Ductal carcinoma in situ
DMFS: Distant metastasis-free survival
EGFR: Epidermal growth factor receptor
EMT: Epithelial–mesenchymal transition
ER: Estrogen receptor
FOXA1: Forkhead box protein A1
HER2: Human epidermal growth factor receptor 2
HR: Hazard ratio
IBC: Invasive breast cancer
IDC: Invasive ductal carcinoma
IF: Intermediate filament
IHC: Immunohistochemistry
INPP4b: Inositol polyphosphate-4-phosphatase
LVI: Lymph vessel invasion
M: Multivariate analysis
MBC: Metastatic breast cancer
MT1: G-protein-coupled receptor for melatonin
MVD: Microvessel density
Oct-4: Octamer-binding transcription factor 4
OR: Odds ratio
OS: Overall survival
PFS: Progression-free survival
PR: Progesterone receptor
qPRT-PCR: Quantitative real time polymerase chain reaction
RCT: Randomized controlled trial
RFS: Recurrence-free survival
TNBC: Triple-negative breast cancer
TNM: Tumor node metastasis
U: Univariate analysis
VPI: Vascular proliferation index
