# Supplementary material for: Clinicopathological and prognostic significance of nestin expression in patients with breast cancer: a systematic review and meta-analysis
Source: Cancer Cell Int. 2020 May 14;20:169. doi: 10.1186/s12935-020-01252-5 (PMC7227264; doi:10.1186/s12935-020-01252-5)
Supplement: Supplementary file 1 — Additional file 1: Table S1. Newcastle–Ottawa Scale for assessing the quality of studies in quantitative analysis. [file 12935_2020_1252_MOESM1_ESM.docx]

## Additional file

## Table S1. Newcastle-Ottawa Scale for assessing the quality of studies in quantitative analysis

| Study ID | Year | Representativeness of the exposed cohort | Selection of the non-exposed cohort | Ascertainment of exposure | Demonstration that outcome of interest was not present at start of study | Comparability of cohorts on the basis of the design or analysis | Assessment of outcome | Was follow-up long enough for outcomes to occur | | Adequacy of follow-up of cohorts | Total scores |  |
| --- | --- | --- | --- | --- | --- | --- | --- | --- | --- | --- | --- | --- |
| Asleh K*, et al* | 2018 | ★ | ★ | ★ | ★ | ★☆ | ★ | | ★ | ★ | 8 | |
| Asleh K, *et al* | 2019 | ★ | ★ | ★ | ★ | ★☆ | ★ | | ★ | ★ | 8 | |
| De Lara S, *et al* | 2019 | ★ | ★ | ★ | ★ | ★☆ | ★ | | ★ | ★ | 8 | |
| Gao NN, *et al* | 2014 | ★ | ★ | ★ | ★ | ★☆ | ★ | | ★ | ★ | 8 | |
| Krüger K, *et al* | 2017 | ★ | ★ | ★ | ★ | ★☆ | ★ | | ★ | ★ | 8 | |
| Liu CG, *et al* | 2010 | ★ | ★ | ★ | ★ | ★☆ | ★ | | ★ | ★ | 8 | |
| Liu CG, *et al* | 2012 | ★ | ★ | ★ | ★ | ☆☆ | ★ | | ★ | ★ | 7 | |
| Meisen WH, *et al* | 2014 | ★ | ★ | ★ | ★ | ☆☆ | ★ | | ★ | ★ | 7 | |
| Nowak A, *et al* | 2017 | ★ | ★ | ★ | ★ | ☆☆ | ★ | | ★ | ★ | 7 | |
| Nowak A, *et al* | 2018 | ★ | ★ | ★ | ★ | ☆☆ | ★ | | ★ | ★ | 7 | |
| Parry S, *et al* | 2008 | ★ | ★ | ★ | ★ | ★☆ | ★ | | ★ | ★ | 8 | |
| Piras F, *et al* | 2011 | ★ | ★ | ★ | ★ | ★☆ | ★ | | ★ | ★ | 8 | |
| Tampaki EC, *et al* | 2017 | ★ | ★ | ★ | ★ | ☆☆ | ★ | | ★ | ★ | 7 | |
| Zhao ZW, *et al* | 2014 | ★ | ★ | ★ | ★ | ☆☆ | ★ | | ★ | ★ | 7 | |
| ☆: scored 0, ★: scored 1. | | | | | | | | | | | |  |
