# Supplementary figures and images for: Clinicopathological and prognostic significance of nestin expression in patients with breast cancer: a systematic review and meta-analysis
Source: Cancer Cell Int. 2020 May 14;20:169. doi: 10.1186/s12935-020-01252-5 (PMC7227264; doi:10.1186/s12935-020-01252-5)

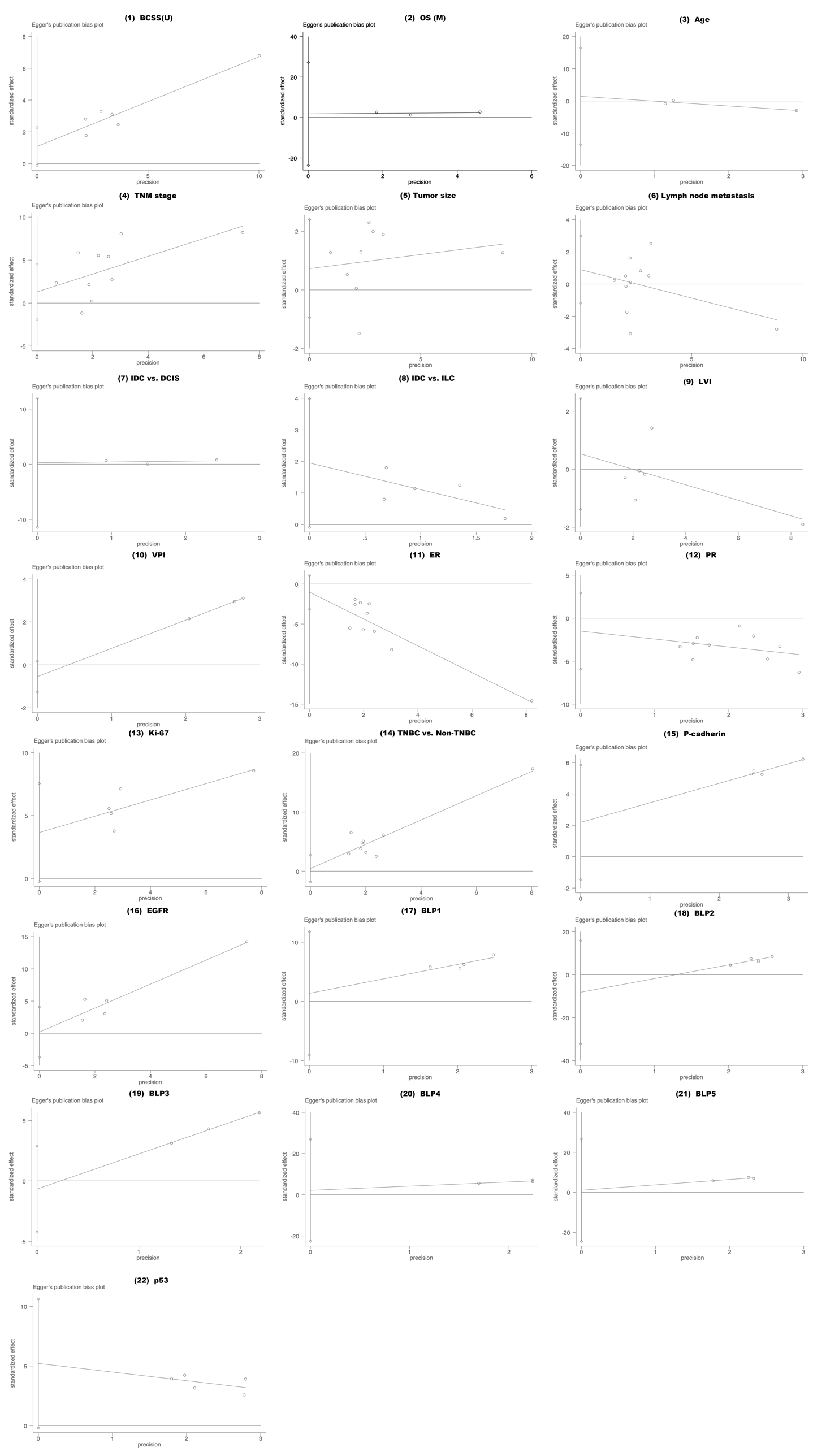

Supplement: Supplementary file 4 — Additional file 4: Fig. S3. Egger’s test. (1) BCSS (univariate analysis); (2) OS (multivariate analysis); (3) Age (age > 35 vs. age < 35); (4) TNM stage (grade III vs. grade I–II); (5) Tumor size (T2–4 vs. T1); (6) Lymph node metastasis (N+ vs. N0); (7) IDC vs. DCIS; (8) IDC vs. ILC; (9) LVI (positive vs. negative); (10) VPI (high vs. low); (11) ER (positive vs. negative); (12) PR (positive vs. negative); (13) Ki-67 (high vs. low); (14) TNBC vs. Non-TNBC; (15) P-cadherin (positive vs. negative); (16) EGFR (positive vs. negative); (17) BLP1 (present vs. absent); (18) BLP2 (present vs. absent); (19) BLP3 (present vs. absent); (20) BLP4 (present vs. absent); (21) BLP5 (present vs. absent); (22) p53 (positive vs. negative). [file 12935_2020_1252_MOESM4_ESM.jpg]
